# Sarcopenia in subjects with Alzheimer’s disease: prevalence and comparison of agreement between EGWSOP1, EGWSOP2, and FNIH criteria

**DOI:** 10.1186/s12877-024-04890-w

**Published:** 2024-03-22

**Authors:** Roberta Barone, Giulia Bramato, Valentina Gnoni, Alessia Giugno, Daniele Urso, Chiara Zecca, Salvatore Nigro, Marco Filardi, Giancarlo Logroscino

**Affiliations:** 1 Center for Neurodegenerative Diseases and the Aging Brain, University of Bari Aldo Moro at Pia Fondazione “Card. G. Panico”, Tricase, Italy; 2 Department of Translational Biomedicine and Neurosciences (DiBraiN), University of Bari Aldo Moro, Bari, Italy

**Keywords:** Sarcopenia, Prevalence, Alzheimer’s disease, Muscle strength, Muscle mass

## Abstract

**Background:**

Sarcopenia is an age-related clinical syndrome characterized by the progressive loss of muscle mass and muscle strength. It appears to be closely linked to dementia, particularly Alzheimer’s disease (AD); however, its prevalence among AD patients remains unclear. In this study, we assessed differences in sarcopenia prevalence between non-demented individuals and AD patients. Moreover, we assessed sex-specific differences in sarcopenia prevalence and explored the diagnostic value of the Muscle Quality Index (MQI) for diagnosing sarcopenia among AD patients.

**Method:**

Cross-sectional study including 145 patients with probable AD and 51 older adults with normal cognition. Sarcopenia was diagnosed according to the criteria of the European Working Group on Sarcopenia in Older People (EWGSOP1 and EWGSOP2) and of the Foundation for the National Institutes of Health (FNIH). The MQI was computed as the ratio of handgrip strength to skeletal muscle mass.

**Results:**

No significant difference in sarcopenia prevalence was observed between AD patients and controls. Prevalence ranged from 3.4 to 23.4% in AD patients and from 2 to 11.8% in controls, depending on diagnostic criteria. Prevalence was higher using EWGSOP1 and decreased using EWGSOP2 and FNIH. Prevalence was higher in males than in females with AD. The MQI was lower in AD patients than in controls (95%CI: − 0.23, − 0.05, *p* < 0.001), but displayed poor diagnostic accuracy in identifying sarcopenia cases.

**Conclusions:**

AD patients and controls show comparable sarcopenia prevalence. Sarcopenia prevalence is higher in males than females among AD patients and higher when using EWGSOP1 compared to FNIH and EWGSOP2 criteria.

## Introduction

Sarcopenia is a complex age-related clinical syndrome characterized by the progressive loss of muscle mass, muscle strength, and/or physical performance, which is associated with considerable morbidity and healthcare costs [1, 2]. It is estimated to affect 10-16% of older adults worldwide [3], with a higher prevalence among nursing-home residents and hospitalized individuals [4]. Sarcopenia is closely linked to dementia, particularly Alzheimer’s disease (AD) [5]. Longitudinal studies on non-demented older adults have shown that a more severe sarcopenia at baseline is associated with a higher risk of incident AD [6]; nevertheless, the question of whether sarcopenia is more prevalent in AD patients compared to the general population remains unclear.

Obtaining unbiased prevalence estimates of sarcopenia in AD has proven challenging, as the limited studies on this topic have reported widely different prevalence rates, ranging from 23.3 to 68% [7–11]. This variability in prevalence rates may be attributed to the diverse ages of the AD patients under investigation and/or to the heterogeneous diagnostic criteria used to identify cases.

Currently, three different diagnostic criteria are commonly used to diagnose sarcopenia in non-Asian subjects: the European Working Group on Sarcopenia in Older People criteria of 2010 (EWGSOP1) [12], its revised version of 2018 (EWGSOP2) [13], and the Foundation for the National Institutes of Health criteria (FNIH) [14]. All the aforementioned criteria require documentation of a reduction in muscle mass, muscle strength, and/or physical performance to establish diagnosis; however, significant differences exist among these criteria regarding which features should be considered the primary indicator of sarcopenia, as well as in the cut-off values used to objectively identify the reduction of muscle mass and muscle strength. Nevertheless, recent evidence from longitudinal studies has highlighted that considering muscle mass and muscle strength independently in the assessment of sarcopenia may be misleading, as these aspects are not strictly parallel, with reduction of muscle strength occurring significantly more rapidly than the concurrent reduction of muscle mass [15].

In this scenario, muscle quality, defined as the amount of strength per unit of muscle mass and quantified by the muscle quality index (MQI) [16], has recently been proposed as a novel index that could potentially serve as a more reliable indicator of sarcopenia compared to muscle strength or muscle mass alone [17]. Nonetheless, studies that have explored the potential diagnostic utility of the MQI in identifying sarcopenia among AD patients are lacking.

This study aims to investigate differences in sarcopenia prevalence, diagnosed according to three different criteria, between AD patients and non-demented older adults. As secondary aims we explored sex-specific differences in sarcopenia prevalence among AD patients and investigated the potential diagnostic utility of the MQI in identifying sarcopenia cases.

## Methods

### Participants

We enrolled one hundred forty-five consecutive patients with mild or major neurocognitive disorder due to AD, referred to the Center for Neurodegenerative Diseases and the Aging Brain of the University of Bari Aldo Moro in Tricase. Diagnosis was established based on the Diagnostic and Statistical Manual of Mental Disorders, Fifth Edition criteria [18]. All subjects underwent a standardized diagnostic protocol, which included neurological and neuropsychological assessment, brain MRI, and lumbar puncture to assay biomarker of neurodegeneration in the cerebrospinal fluid [19]. The control group consists of fifty-one non-demented individuals referred to our center for suspected neurological disorders and/or complaints of cognitive decline, who, however, showed no objective neuropsychological deficits and had unremarkable clinical, neuroimaging, and fluid biomarker examinations [20]. The experimental protocol was approved by the ethics committee of ASL Lecce (verbale n°6, July 25th, 2017) and conducted in accordance with the Declaration of Helsinki. Written informed consent was obtained from all participants.

### Nutritional and anthropometric assessment

All participants underwent a comprehensive nutritional evaluation conducted by an experienced nutritionist with expertise in neurodegenerative diseases. Handgrip strength (HGS) was assessed using a hand dynamometer (DynX dynamometer, Akern s.r.l., Montacchiello, Italy). HGS was measured twice for each hand, and the highest score was recorded and used for analysis.

Body composition was assessed through bioelectrical impedance analysis (BIA) (BIA101 Anniversary, Akern s.r.l., Montacchiello, Italy). BIA was performed in a room with a temperature between 24 and 26 °C, with the subjects lying supine after 15 minutes of rest. Electrodes were positioned on the right hand and foot, and participants were instructed to abstain from consuming alcoholic beverages and engaging in excessive exercise for 24 hours prior to the evaluation. The following BIA metrics were considered: body mass index (BMI), skeletal muscle mass (SM, which accounts for 40% of total body weight), appendicular skeletal muscle mass (ASM, sum of the muscle mass of four limbs), and skeletal muscle index (SMI, SM divided by height squared). The MQI was computed as the ratio of HGS to SM [16].

### Sarcopenia diagnostic criteria

Sarcopenia was diagnosed based on the EWGSOP1, EWGSOP2 and FNIH criteria.

According to EWGSOP1, the primary indicator of sarcopenia is a reduction in muscle mass (SMI < 10.76 *kg/m*^2^ for men and < 6.76 *kg/m*^2^ for woman), and a concomitant reduction in muscle strength (HGS < 30 *kg* for men and < 20 *kg* for woman) is required for diagnosis [12].

EWGSOP2 considers a reduction in muscle strength (HGS < 27 *kg* for men and < 16 *kg* for woman) as the primary indicator of sarcopenia, requiring a concomitant reduction in muscle mass **(**ASM/height^2^ < 7 *kg/m*^*2*^ for men and < 5.5 *kg/m*^*2*^ for woman**)** for diagnosis [13].

 Similarly, FNIH considers a reduction in muscle strength (HGS < 26 *kg* for men and < 16 *kg* for woman) as a primary indicator of sarcopenia and requires a concomitant reduction in muscle mass (ASM/BMI < 0.789 *kg* for men and < 0.512 *kg* for woman) to establish diagnosis [14].

### Statistical analysis

Data were explored using descriptive statistics (mean ± standard deviation or frequency).

Differences in age, sex distribution, and BMI between AD patients and controls were analyzed by means of Student’s t-test and chi-squared test. Differences in HGS and muscle mass metrics between AD patients and controls were analyzed through Student’s t-test corrected for age, sex, and BMI. The strength of the association between sarcopenia and AD was evaluated, separately for each diagnostic criterion, through logistic regression analyses adjusted for age, sex, and BMI. The diagnostic agreement between EWGSOP1, EWGSOP2 and FNIH criteria was assessed using Cohen’s kappa (*k*) [21]. Receiver operating characteristic curve (ROC) analysis was used to evaluate the diagnostic utility of the MQI in identifying sarcopenia as diagnosed by EWGSOP1, EWGSOP2 and FNIH criteria. Differences in demographic, clinical data and in sarcopenia prevalence between males and females AD patients were analyzed through chi-squared test and Student’s t-test corrected for age and BMI. Finally, AD patients were classified as mild, moderate, or severe based on their MMSE score (between 23 and 21, 20-12 and < 12, respectively) [22], and between-group differences in HSG, muscle mass metrics, and sarcopenia prevalence were analyzed through of analysis of variance corrected for age, sex, and BMI. Statistical analyses were conducted using SPSS 19.0 (SPSS, Inc. Chicago, IL) and results with *p*-values < 0.05 were considered statistically significant.

## Results

Differences in demographic, clinical data, and sarcopenia prevalence between AD patients and controls, along with the corresponding test statistic (t or χ^2^) and *p*-values, are reported in Table 1. AD patients were significantly older (t_(194)_: 4.60, *p* < 0.0001) and presented with lower BMI (t_(194)_: − 2.61, *p* < 0.01) and MMSE scores (t_(194)_: − 13.72, *p* < 0.0001) than controls. No significant group difference emerged in sex distribution (χ^2^: 0.13, *p* = ns). After controlling for age, sex and BMI, significant differences were observed in HGS and MQI, with AD patients showing lower HGS (*p* < 0.0005) and MQI (*p* < 0.001) compared to controls. No difference emerged in sarcopenia prevalence between AD patients and controls (all *p* = ns). Sarcopenia prevalence in AD patients was 23.4% according to EWGSOP1, 3.4% according to EWGSOP2, and 9.7% according to FNIH, while in controls it was 11.8% according to EWGSOP1, 2% according to EWGSOP2, and 5.9% according to FNIH. AD patients were more likely than controls to present with reduced muscle strength as defined by EWGSOP1 (χ^2^: 12.96, *p* < 0.0005), EWGSOP2 (χ^2^: 14.12, *p* < 0.0005), and FNIH criteria (χ^2^: 11.53, *p* < 0.001). Logistic regression analysis did not any reveal significant association between sarcopenia diagnosed according to EGWSOP1 (OR: 1.97, 95%CI: 0.62, 3.19, *p* = 0.25), EGWSOP2 (OR: 1.03, 95%CI: 0.10, 9.89, *p* = 0.98), or FNIH criteria (OR: 4.01, 95%CI: 0.77, 20.83, *p* = 0.10) and AD. Regarding the diagnostic agreement between sarcopenia criteria, EWGSOP1 showed fair agreement with FNIH (Cohen’s *k*: 0.38) and slight agreement with EWGSOP2 (Cohen’s *k*: 0.22) criteria. EWGSOP2 and FNIH showed fair agreement (Cohen’s *k*: 0.32). The MQI showed an area under the ROC curve of 0.53 (95%CI: 0.43, 0.62) for sarcopenia diagnosed according to EWGSOP1, and AUCs of 0.35 (95%CI: 0.13, 0.57) and of 0.52 (95%CI: 0.39, 0.65) for sarcopenia diagnosed according to EWGSOP2 and FNIH criteria, respectively.

**Table 1:** Demographic, clinical data, HGS, muscle mass metrics and sarcopenia prevalence rates in AD patients and controls

	AD (*n* = 145) Mean ± SD	Controls (*n* = 51) Mean ± SD	t_(194) or_ χ^2^	*p*-values
*Demographic and clinical data*				
Male/Female	61/84	20/31	0.13	0.722
Age, *y*	71.51 ± 8.31	64.71 ± 11.02	4.60	<0.0001
BMI	25.88 ± 3.96	27.65 ± 4.75	−2.61	<0.01
MMSE	16.92 ± 5.39	27.51 ± 1.89	−13.72	<0.0001
*Muscle Strength and Muscle Mass*				
HGS	17.47 ± 7.22	21.78 ± 8.30	13.93^a^	<0.0005
SMI	9.73 ± 1.59	9.98 ± 1.75	0.01^a^	0.938
SM	26.38 ± 5.31	25.75 ± 6.27	0.17^a^	0.683
ASM	20.85 ± 4.08	19.88 ± 4.10	0.07^a^	0.790
ASM/height^2^	7.54 ± 0.97	7.88 ± 1.28	0.00^a^	0.956
MQI	0.70 ± 0.29	0.84 ± 0.28	11.33^a^	<0.001
*Sarcopenia diagnostic algorithms*				
EWGSOP1, Low muscle mass (%)	37 (25.5%)	9 (17.6%)	1.30	0.254
EWGSOP1, Low muscle strength (%)	119 (82.1%)	29 (56.9%)	12.96	<0.0005
EWGSOP1, Sarcopenia (%)	34 (23.4%)	6 (11.8%)	3.17	0.075
EWGSOP2, Low muscle strength (%)	95 (65.5%)	18 (35.3%)	14.12	<0.0005
EWGSOP2, Low muscle mass (%)	6 (4.1%)	3 (5.9%)	0.26	0.609
EWGSOP2, Sarcopenia (%)	5 (3.4%)	1 (2.0%)	0.28	0.596
FNIH, Low muscle strength (%)	91 (62.8%)	18 (35.3%)	11.53	<0.001
FNIH, Low muscle mass (%)	18 (12.4%)	10 (19.6%)	1.59	0.207
FNIH, Sarcopenia (%)	14 (9.7%)	3 (5.9%)	0.68	0.410

*AD* Alzheimer disease, *BMI* Body Mass Index, *HGS*: hand grip strength, *SMI* Skeletal Muscle Index, *SM* Skeletal Muscle Mass, *ASM* Appendicular Skeletal Muscle Mass, *MQI* Muscle Quality Index, *EWGSOP*1 European Working Group on Sarcopenia in Older People criteria of 2010, *EWGSOP*2 European Working Group on Sarcopenia in Older People criteria of 2018, *FNIH* Foundation for the National Institutes of Health criteria

^a^Student’s t-test corrected for age, sex and BMI

Differences in demographic, clinical data, and sarcopenia prevalence rates between male and female AD patients are reported in Table 2. Male AD patients showed higher HGS, SMI, SM, ASM and ASM/height^2^ compared to female patients (all *p* < 0.0001). Sarcopenia prevalence was higher in male AD patients than in females according to all diagnostic criteria (EWGSOP1:55.7% vs 0%; EWGSOP2: 8.2% vs 0%; FNIH: 19.7% vs 2.4%). Furthermore, male AD patients were more likely to present reduced muscle mass, as defined by EWGSOP1 (χ^2^: 62.17, *p* < 0.0001), EWGSOP2 (χ^2^: 4.37, *p* < 0.05), and FNIH criteria (χ^2^: 7.67, *p* < 0.01), than female AD patients. Similar results were observed in controls, with a higher percentage of males meeting EWGSOP1 (6 vs 0, χ^2^: 10.54, *p* < 0.001) and FNIH (3 vs 0, χ^2^: 4.94, *p* < 0.05) criteria for sarcopenia compared to females, while no difference emerged regarding EWGSOP2 criteria (1 vs 0, χ^2^: 1.58, *p* = ns). There was no significant difference in the MQI between male and female AD patients (t_(143)_: 0.01, *p* = ns).

**Table 2:** Demographic, clinical data, HGS, muscle mass metrics and sarcopenia prevalence rates in male and female AD patients

	AD male (*n* = 61) Mean ± SD	AD female (*n* = 84) Mean ± SD	t_(143) or_ χ^2^	*p*-values
*Demographic and clinical data*				
Age, *y*	70.57 ± 9.09	72.19 ± 7.68	−1.16	0.249
BMI	25.46 ± 3.02	26.18 ± 4.51	−1.03	0.280
MMSE	16.57 ± 5.37	17.17 ± 5.42	−0.65	0.515
*Muscle Strength and Muscle Mass*				
HGS	21.36 ± 7.26	14.64 ± 5.74	37.39^a^	<0.0001
SMI	10.77 ± 1.27	8.97 ± 1.36	67.83^a^	<0.0001
SM	30.96 ± 4.84	21.96 ± 4.11	152.22^a^	<0.0001
ASM	23.03 ± 3.48	17.60 ± 2.79	136.95^a^	<0.0001
ASM/height^2^	8.01 ± 0.87	7.20 ± 0.90	48.51^a^	<0.0001
MQI	0.70 ± 0.23	0.70 ± 0.32	0.01^a^	0.907
*Sarcopenia diagnostic algorithms*				
EWGSOP1, Low muscle mass (%)	36 (59%)	1 (1.2%)	62.17	<0.0001
EWGSOP1, Low muscle strength (%)	54 (88.5%)	65 (77.4%)	2.98	0.084
EWGSOP1, Sarcopenia (%)	34 (55.7%)	0	61.16	<0.0001
EWGSOP2, Low muscle strength (%)	47 (77%)	48 (57.1%)	6.20	<0.005
EWGSOP2, Low muscle mass (%)	5 (8.2%)	1 (1.2%)	4.37	<0.05
EWGSOP2, Sarcopenia (%)	5 (8.2%)	0	7.13	<0.01
FNIH, Low muscle strength (%)	43 (70.5%)	48 (57.1%)	2.69	0.101
FNIH, Low muscle mass (%)	13 (21.3%)	5 (6%)	7.67	<0.01
FNIH, Sarcopenia (%)	12 (19.7%)	2 (2.4%)	12.11	<0.001

*AD* Alzheimer disease, *BMI* Body Mass Index, *HGS* hand grip strength, *SMI* Skeletal Muscle Index, *SM* Skeletal Muscle Mass, *ASM* Appendicular Skeletal Muscle Mass, *MQI* Muscle Quality Index, *EWGSOP*1 European Working Group on Sarcopenia in Older People criteria of 2010, *EWGSOP*2 European Working Group on Sarcopenia in Older People criteria of 2018, *FNIH* Foundation for the National Institutes of Health criteria

^a^Student’s t-test corrected for age and BMI

After controlling for the effects of age and BMI, no significant differences were observed in HGS, SMI, SM, ASM, ASM/height^2^ and MQI, between mild, moderate, and severe AD patients (all *p* = ns).

**Fig. 1:**
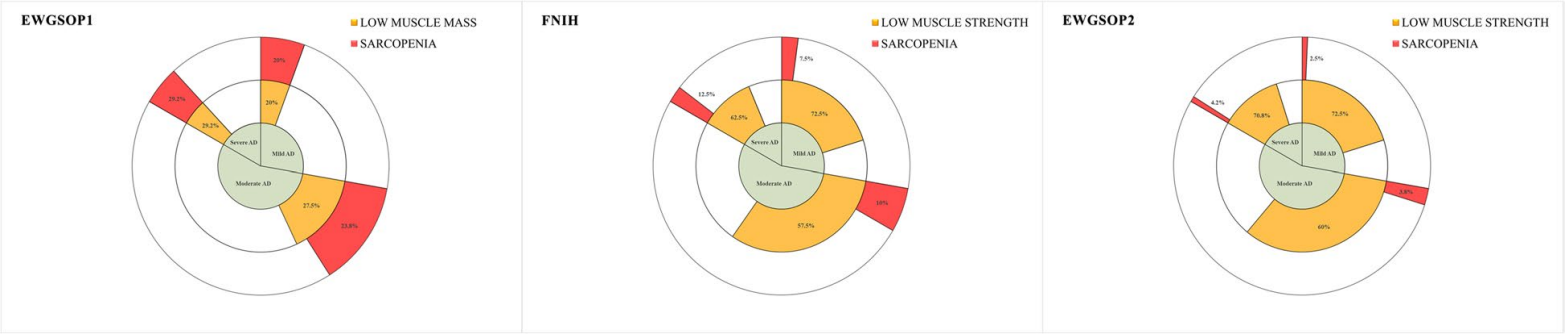
Sarcopenia prevalence rates according to EWGSOP1, FNIH and EWGSOP2 criteria in mild, moderate, and severe AD patients

The prevalence of sarcopenia in mild, moderate, and severe AD patients is depicted in Fig. 1.

According to EWGSOP1, sarcopenia was present in 20% of mild AD, 23.8% of moderate AD and 29.2% of severe AD patients (*χ*^*2*^: 0.70, *p* = ns). A similar trend was observed for both FNIH (7.5% in mild AD, 10% in moderate AD, and 12.5% in severe AD patients, *χ*^*2*^: 0.44, *p* = ns) and EWGSOP2 criteria (2.5% in mild AD, 3.8% in moderate AD and 4.2% in severe AD patients, *χ*^*2*^: 0.166, *p* = ns).

## Discussion

In this study, we assessed differences in sarcopenia prevalence, diagnosed according to the three different criteria, between AD patients and non-demented older adults.

We found no significant difference in sarcopenia prevalence between AD patients and controls, with rates ranging from 3.4 to 23.4% in AD patients and from 2 to 11.8% in controls.

Overall, the prevalence of sarcopenia observed in our sample of AD patients aligns with results of previous studies on sarcopenia prevalence in AD patients of comparable age.

The study by Lee et al. reported a sarcopenia prevalence of 29.6% (sarcopenia diagnosed according to EGWSOP1 criteria) in a sample of mild-to-moderate AD patients in their 70s [7]. Comparable prevalence rates were observed in the studies by Sugimoto et al. and Hirose et al., who reported sarcopenia rates of 23.3 and 30% (sarcopenia diagnosed according to the Asian Working Group for Sarcopenia criteria, AWGS), respectively, among AD patients of a similar age [8, 9].

Conversely, studies that investigated sarcopenia prevalence among older AD patients (i.e., > 80 years) have consistently reported higher prevalence rates. The study by Ogawa et al. reported a sarcopenia prevalence of 45.55% in mild AD patients and 55.75% in moderate AD patients (EGWSOP1 criteria) [10]. Similarly, the studies by Demura et al. and Hirose et al. reported a sarcopenia prevalence of 62.8 and 68%, respectively (AWGS criteria) [9, 11].

Regarding differences in the estimated prevalence of sarcopenia according to different diagnostic criteria, to the best of our knowledge, this is the first study to address this topic in AD patients.

The prevalence of sarcopenia was 23.4% when using EWGSOP1 and decreased to 9.7 and 3.4% when applying FNIH and EWGSOP2 criteria, respectively. This finding is consistent with results of previous studies on sarcopenia prevalence among non-demented older adults, which showed that, regardless of demographic variables such as ethnicity, sex, and age range, EWGSOP2 diagnoses fewer older adults with sarcopenia than EWGSOP1 [23, 24]. Furthermore, the analysis of inter-criteria agreement revealed a poor agreement among sarcopenia criteria, with fair-to-slight agreement between EWGSOP1, EWGSOP2 and FNIH (Cohen’s *k*: 0.22-0.38), and a fair agreement between EWGSOP2 and FNIH (Cohen’s *k*: 0.32). Comparable results have been reported in previous studies on non-demented community-dwelling older adults, which revealed poor or null agreement between sarcopenia criteria [25, 26]. Several factors could contribute to the discrepancy in sarcopenia case identification among EWGSOP1, EWGSOP2, and FNIH. EWGSOP1 considers the reduction of muscle mass as the primary indicator of sarcopenia, whereas both EWGSOP2 and FNIH prioritize the reduction of muscle strength over muscle mass. Moreover, the cut-off values for muscle mass and muscle strength vary across criteria, with EWGSOP2 using more stringent cutoffs than EWGSOP1, and FNIH criteria considering BMI, rather than height, to adjust muscle mass for body size despite using the same cutoff values.

Concerning the single-dimensional aspects of sarcopenia (i.e., reduced muscle mass and muscle strength), we did not find significant differences in the muscle mass metrics considered by sarcopenia diagnostic criteria (i.e., SMI, ASM and ASM/height^2^) between AD patients and controls. Conversely, handgrip strength was significantly lower in AD patients compared to controls, consistent with findings from previous studies [19, 27]. Finally, we found that the MQI was lower in AD patients than in controls but showed poor diagnostic accuracy (AUC ranging between 0.35 and 0.53) in identifying sarcopenia cases, regardless of the diagnostic criteria adopted. This result does not support the conclusion that the MQI could be used to reliably assess sarcopenia among AD patients, although further studies are necessary before drawing definitive conclusions.

Regarding sex-specific differences in sarcopenia prevalence, we found that male AD patients showed significantly higher sarcopenia prevalence than females, according to all diagnostic criteria considered. The same trend of difference was observed in the controls group.

Several epidemiological studies have reported a gender discrepancy in sarcopenia prevalence, however whether sarcopenia is disproportionately more prevalent in older women or men remains unclear [28–33]. In the field of AD, to date, only the study by Ogawa et al. has explored sex-specific difference in sarcopenia prevalence, reporting no significant difference in prevalence rates between male and female AD patients (49.17% vs 45.10%, respectively) [10]. Although the sex-specific pathophysiological mechanisms underlying sarcopenia are still not fully understood, recent studies have suggested that sex hormones may, at least in part, contribute to the observed differences in sarcopenia rates between genders. Sex hormones undergo an age-related decline and display a crucial role in skeletal muscle metabolism, influencing insulin resistance, lipid substrate usage, anabolic hormones, and catabolic markers [34, 35].

Finally, concerning differences in sarcopenia prevalence between mild, moderate, and severe AD patients we observed a non-significant increase in prevalence across the different clinical stages of AD (mild AD: 2.5-20%, moderate AD: 3.8-23.8%, severe AD: 4.2-29.2%). A similar trend was reported in the study by Ogawa et al., who analyzed an older sample of AD patients and found that sarcopenia prevalence was 36.5% in early AD, 45.6% in mild AD, and 55.8% in severe AD patients [10]. Over the last decade, there has been increasing recognition of a link between sarcopenia and an increased risk of AD, as well as a more rapid rate of cognitive decline [36].

One of the main factors contributing to sarcopenia likely stems from the diminished input of α-motor neurons to the muscle, given the essential role of muscle innervation in maintaining muscle mass and strength [37]. As individuals age, there is a decline in the number of operational motor units, coupled with an enlargement of the cross-sectional area of the remaining units, resulting in a reduction in both fibers and operational motor units, as well as an enlargement in motor unit size [38]. Although the loss of α-motor neuron input is not typically associated with AD, AD pathology may indirectly impact motor function by disrupting neural systems crucial for the sequential or parallel processes necessary for movement planning and execution [39]. Additionally, other candidate mechanisms such as insufficient exercise and dietary intake, poor sleep quality, age-related hormonal changes, inflammatory abnormalities, and oxidative stress, have been proposed to explain the association between sarcopenia and AD [40, 41]. Nonetheless, whether the pathophysiology of sarcopenia differs between patients with AD and older adults is still unclear, and further studies on this topic are highly warranted. Some limitations of this study should be acknowledged. Firstly, AD patients were significantly older than controls, due to the difficulty in enrolling patients over 65 years of age without cognitive impairments among subjects referred to a center specialized in neurodegenerative diseases. Although the age difference has been taken into account in the statistical analysis, it could still exert a significant influence on the results, particularly regarding sarcopenia prevalence. Moreover, the control group is relatively small compared to AD patients. Second, physical performance was not routinely assessed at the times of data collection. This issue could have led to an underestimation of sarcopenia prevalence as per EWGSOP1 criteria and precluded us from identifying severe sarcopenia according to EWGSOP2 criteria. Finally, given the overall low prevalence of sarcopenia among our sample of AD patients, the analysis of MQI diagnostic performance might be underpowered and further studies are necessary before drawing definitive conclusions about the diagnostic usefulness of the MQI in AD.

## Conclusions

Patients with AD and controls show comparable sarcopenia prevalence. The prevalence of sarcopenia is higher in male than female AD patients and increases when using EWGSOP1 compared to FNIH and EWGSOP2 criteria. Diagnostic criteria show fair-to-slight agreement in identifying sarcopenia, emphasizing the need to develop specific criteria to enhance the recognition of sarcopenia in AD patients.

## Data Availability

The dataset supporting the conclusions of this article is available from the corresponding author upon reasonable request.

## Abbreviations

AD: Alzheimer’s disease
EWGSOP1: European Working Group on Sarcopenia in Older People of 2010 criteria
EWGSOP2: European Working Group on Sarcopenia in Older People of 2019 criteria
FNIH: Foundation for the National Institutes of Health criteria
MQI: Muscle quality index
DSM-5: Diagnostic and Statistical Manual of Mental Disorders, fifth edition
MMSE: Mini-Mental State Examination
HGS: Handgrip strength
BIA: Bioelectrical impedance analysis
BMI: Body mass index
SM: Skeletal muscle mass
ASM: Appendicular skeletal muscle mass
SMI: Skeletal muscle index
ROC: Receiver operating characteristic
AUC: Area under the curve
AWGS: Asian Working Group for Sarcopenia criteria
